# Quasi-radial growth of metal tube on si nanowires template

**DOI:** 10.1186/1556-276X-6-165

**Published:** 2011-02-23

**Authors:** Zhipeng Huang, Lifeng Liu, Nadine Geyer

**Affiliations:** 1 Functional Molecular Materials Centre, Scientific Research Academy, Jiangsu University, Zhenjiang 212013, P. R. China; 2 Max Planck Institute of Microstructure Physics, Weinberg 2, D-06120 Halle/Saale, Germany

## Abstract

It is reported in this article that Si nanowires can be employed as a positive template for the controllable electrochemical deposition of noble metal tube. The deposited tube exhibits good crystallinity. Scanning electron microscope and transmission electron microscope characterizations are conducted to reveal the growth process of metal tube, showing that the metal tube grows quasi-radially on the wall of Si nanowire. The quasi-radial growth of metal enables the fabrication of thickness-defined metal tube via changing deposition time. Inner-diameter-defined metal tube is achieved by choosing Si nanowires with desired diameter as a template. Metal tubes with inner diameters ranging from 1 μm to sub-50 nm are fabricated.

## Introduction

Owing to a considerably enhanced surface-to-volume ratio compared to bulk, one-dimensional metallic tubular structure has shown promising application potential in the fields of energy storage and conversion [1,2], catalysis [3-5], and magnetism [6,7], and therefore has gained increasing attention. Similar to the case of other nanostructures, controllable fabrication is essential for the device application of tubular structure. Various approaches (e.g., electrochemical deposition [8-10], electroless deposition [11,12]), etc., have been developed to fabricate metal tubes. Meanwhile, templates with specific aspect ratio and packing manner are used to define the geometries of nanotubes. Nowadays, two insulating masks, namely, porous anodic aluminum oxide (AAO) and ion-track-etched polymer membrane, are widely used for the fabrication of nanotubes. However, chemical modification (introducing molecular anchor) of pore wall [9,13,14] or metal pre-deposition (as seed layer) on pore wall [12,15] is necessary before the fabrication of metal tube, which will inevitably introduce impurity to the deposited structures [12]. On the other hand, during electrochemical deposition, metal grows along axial direction in the isolating template [8], which makes it difficult for controlling independently the thickness and length of tubular structure. From these points of view, conducting or semi-conducting template is more favorable for the fabrication of metal tube, because the modification of template surface is unnecessary and the growth is hopefully radial. Macroporous silicon (Si) [16-18] and InP [19] have been used as templates for the fabrication of metal tube. However, the feature size in macroporous Si is usually larger than several hundreds of nanometer due to a well-known 2*W*_sc _rule [20], where *W*_sc _is the thickness of space charge layer in Si substrate at Si/solution interface. Moreover, only the tube of less noble metal has been demonstrated on the macroporous Si template, whereas the electrochemical deposition of noble metal leads to wire or pillar, because noble metal grows axially from the bottom of pores in the macroporous Si template [16,17].

Si nanowire would be an alternative candidate as a positive template for the deposition of metal tube, due to its intrinsic semi-conducting property and wide diameter range. Especially, template-based metal-assisted chemical etching [21-25] enables precise control over the diameter, length, orientation relative to substrate, packing manner, and cross-sectional shape of Si nanowires. In this article, it is reported that highly ordered array of Si nanowires fabricated by template-based metal-assisted chemical etching can be used as a positive template for the controllable electrochemical deposition of noble metal (Au) tube. It is indicated by scanning electron microscope (SEM) and transmission electron microscope (TEM) that metal grows quasi-radially on the sidewall of Si nanowire. Therefore, the length and thickness of metal tube can be independently controlled. On the other hand, metal tubes with the inner diameter ranging from 1 μm to sub-50 nm are obtained by choosing Si nanowires with desired diameters as a template.

## Experimental

**Figure 1:**
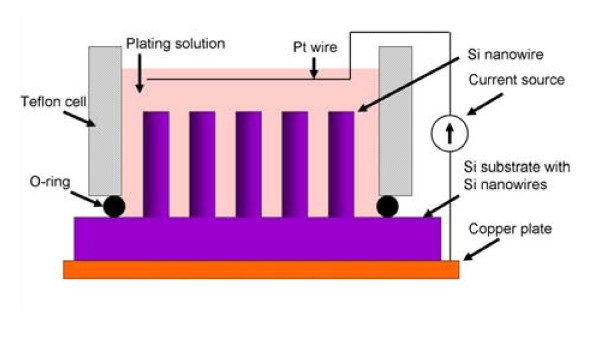
**Schematic illustration showing the experimental setup of electrochemical depositing metal onto Si nanowires**.

**Figure 2:**
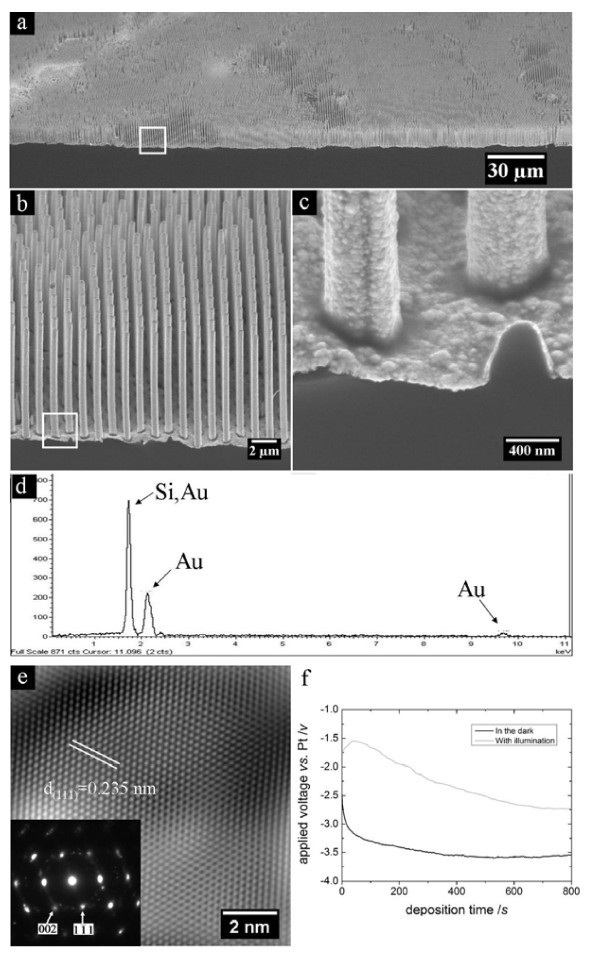
**(a-c) The bird's-eye view of SEM images of Au tube deposited on an ordered array of Si nanowires**. The rectangle in **(a) **encloses a region which is magnified into **(b)**, and the rectangle in **(b) **encloses a region which is magnified into **(c)**. **(d) **EDX spectrum of an Au tube/Si nanowires sample. **(e) **HR-TEM image of an Au tube released from Si nanowire, and (inset of **e**) the [110] zone axis SAED pattern of the Au tube. The white lines indicate projection of atoms on (111) plane along [110] direction. **(f) **Applied potentials versus deposition times for the deposition in the dark (black line) and under room light illumination (gray line), respectively.

**Figure 3:**
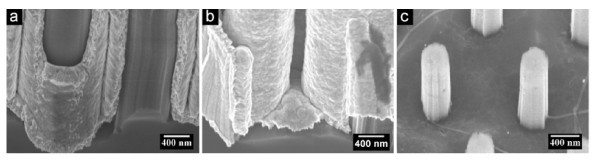
**The bird's-eye view of SEM images of the samples subjected to electrodepositions under the current density of (a) 2 mA/cm^2 ^for 40 min and (b) 1 mA/cm^2 ^for 80 min, respectively, and (c) the sample immersed in the plating solution without applied potential**. The diameters, the lengths, and the inter-wire distances between nanowires of samples used in **(a) **and **(b) **were identical.

**Figure 4:**
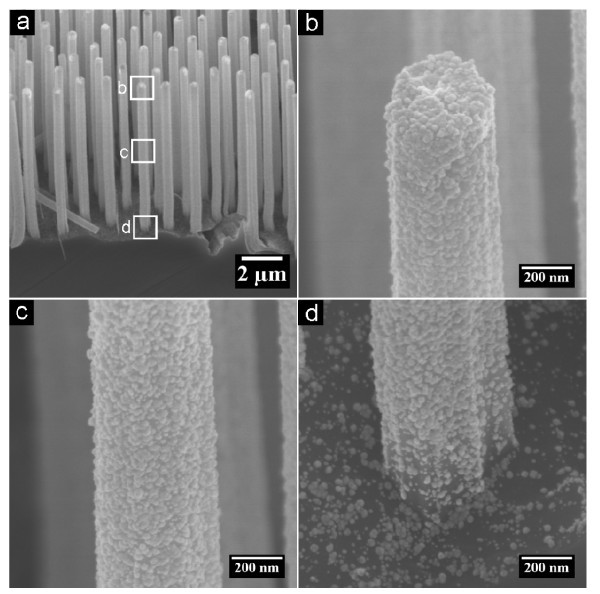
**SEM images of Si nanowires deposited with Au for 5 min**. **(a) **Low magnification image showing the morphologies of the whole wires. **(b-d) **High magnification SEM images showing in detail the morphologies of the top, middle, and root part of a single nanowire, respectively. The rectangles in **(a) **enclose the regions which are magnified into **(b-d)**.

**Figure 5:**
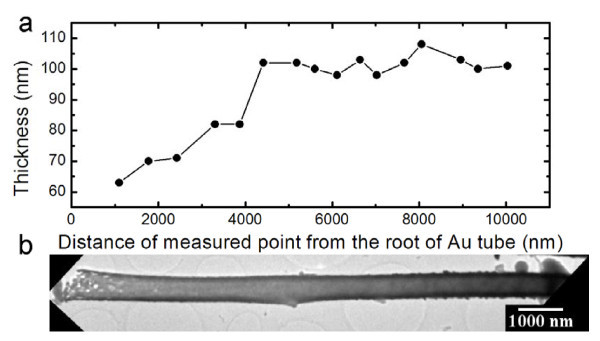
**The thicknesses along a typical Au nanotube**. **(a) **The relationship between the thicknesses of an Au tube and the distances of the measured points from the root of the Au tube. **(b) **Low TEM image of the measured Au tube. The thickness values are measured from higher magnification TEM images.

**Figure 6:**
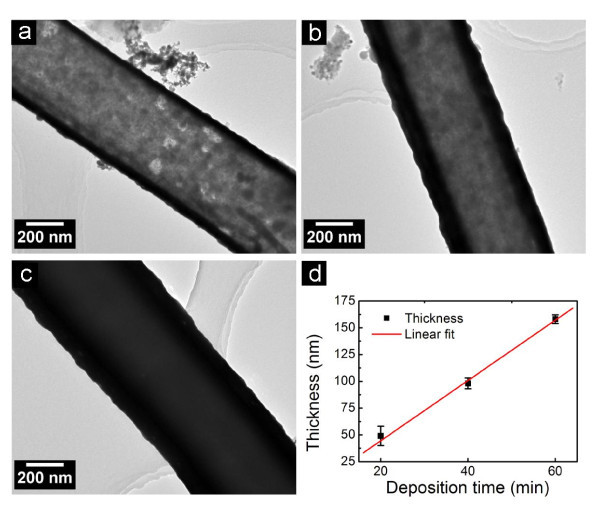
**Typical TEM images of Au tubes deposited with (a) 20 min, (b) 40 min, and (c) 60 min**. **(d) **Relationship between tube thickness and deposition time.

**Figure 7:**
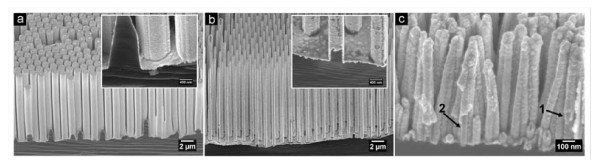
**SEM images of Au tubes deposited on SiNWs with different diameters (a) 1 μm, (b) 450 nm, and (c) 45 nm**. Insets in **(a) **and **(b) **show respective close cross-sectional views revealing the Au tube on Si nanowires. Arrow 1 in **(c) **indicates a broken tube structure. Arrow 2 in **(c) **indicates a Si nanowire template.

Si nanowire templates were fabricated by template-based metal-assisted chemical etching [21,23,24] of Si substrates (ρ: 1-10 Ωcm, *n*-type substrates for samples are shown in Figures 1, 2, 3, 4, 5, 6, 7a and 7b, and *p*-type substrates for samples are shown in Figure 7c). Except the one used in Figure 7, the Si nanowire templates used in this article were fabricated by the metal-assisted chemical etching combined with nanosphere lithography. In brief, polystyrene (PS) spheres were assembled into monolayer hexagonal array onto a Si substrate. Then the diameter of PS spheres was reduced by reactive ion etching. Afterward, a silver (Ag) mesh with ordered pores was obtained by depositing Ag onto the Si substrate with arrays of diameter-reduced PS spheres [21]. Subsequently, the Si substrates loaded with Ag mesh were etched in an etchant composed of HF, H_2_O_2_, and de-ionized water for a certain time. Afterward, the Ag mesh was removed by a 3-min concentrated HNO_3 _treatment, and the Si substrate with Si nanowires was rinsed with copious amount of de-ionized water. For the Si nanowires templates used in Figure 7, AAO membrane was used as template instead of PS sphere for the deposition of Ag mesh, as reported by Huang et al. [23]. The diameter of Si nanowires was defined by the diameter of the pre-defined mask, and the length of Si nanowires was determined by the etching time.

Metal was galvanostatically deposited onto Si nanowires in a two-electrode setup (Figure 1). A home-built Teflon electrochemical cell was used to ensure that only the surface with Si nanowires was exposed to a plating solution. During plating, Si nanowires on a Si substrate acted as a working electrode, and a platinum wire worked as a counter electrode. For the deposition of gold (Au) tube, commercial plating solution (25 mM, Goldplattierbad GP 204, from Heimerle+Meule GmbH, Germany) was used. A Keithley 2400 power supply was used as a current source, and the current density during the deposition was adjusted to 1 mA/cm^2^. The plating experiments were carried out in ambient condition at room temperature. No special attention had to be paid to the contact between backside of Si substrate and Cu electrode. No discernable difference was found between samples plated with and without GaIn eutectic (as an ohmic contact) between Si substrate and Cu plate.

After plating, surface morphologies and element analysis of the Si nanowires with metal tube were characterized by a SEM (JSM 7001F, JEOL) equipped with energy dispersive X-ray analysis system (EDXA, Inca Energy-350, Oxford Instruments, UK). To reveal the thicknesses of tubular structures, TEM (JEM 2100, JEOL) characterization was carried out. For the TEM characterization, the Si substrates with metal tubes were subjected to a concentrated NaOH solution (4.5 M, 50°C, 3 h) to release metal tubes from Si nanowires. Afterward, the metal tubes were extracted via centrifugation, and were rinsed with ethanol until the pH value of solution equaled 7. Finally, the metal tubes/ethanol solution was dropped onto TEM grids.

## Results and discussion

In a typical electrochemical deposition experiment, Au was deposited onto Si nanowires with average diameter of *ca*. 550 nm. During the deposition, a small number of bubbles were observed on the Si nanowire substrate in the electrochemical deposition of Au, which might be due to hydrogen evolution from the Si template. After electrochemical deposition, Au was found to be homogeneously deposited onto the template in a large area, exhibiting bright contrast in SEM images (Figure 2a). The deposited Au film covers fully the side wall of Si nanowires, resulting in Au tube (Figure 2b,c). Interestingly, it is revealed that the Au is deposited not only onto the sidewall of Si nanowire, but also to the plateau between Si nanowires (Figure 2c), implying that the electrochemical deposition uniformly occurred on the entire Si surface irrespective of the surface morphology. It was confirmed by EDXA (Figure 2d) that the deposited film is Au. Au tube deposited on Si nanowire exhibits good crystallinity, as evidenced by the high-resolution TEM (HR-TEM) image (Figure 2e) of an Au tube released from Si nanowire template and the corresponding selected area electron diffraction (SAED) pattern (inset of Figure 2e).

Neither surface modification nor removal of surface Si oxide, which formed because of slow oxidation of as-prepared Si nanowires in the air, was necessary before the electrochemical deposition of Au tubes shown in Figure 2. Control experiments were performed, in which surface oxide was removed by HF-treatment (3.4 wt.%, 5 min) before the electrochemical deposition. The morphologies of Au tubes on Si nanowire templates with or without HF treatment did not exhibit discernable difference. The presence or the absence of surface oxide film is very important in electrochemical deposition. Oxide film of the non-HF treatment templates might have somehow been removed in electrochemical bath. However, it is hard to give solid evidence of oxide removal, because the detail information of commercial available Au plating solution is unknown, and the surface oxide will form again in several minutes in the air even if it was removed by the plating solution during the deposition, introducing difficulty to any *ex situ *TEM characterization.

The depositions were performed in the dark, and under the front-side room light illumination. No discernable morphological difference was found in the resulting Au tubes on corresponding Si templates. The applied potentials during the depositions were recorded, and shown in Figure 2f. The potential necessary for the experiment in the dark is higher than that under illumination. The light irradiating the Si substrate induced photo-generated electron-hole pairs in the template, and the photo-excited electrons could arrive at the Si/solution interface and reduce Au ions because of the applied external potential. Accordingly, only a less applied potential is needed to drive the same amount of electrons to the Si/solution interface in the case of deposition under illumination than in that of deposition in the dark.

The depositions were performed under different current densities. Figure 3a,b shows clearly that the thickness of the deposited Au under 2 mA/cm^2 ^was larger than that under 1 mA/cm^2^, even if the deposition time under 1 mA/cm^2 ^(80 min) was two times of that under 2 mA/cm^2 ^(40 min). The clearance between Si nanowires has been totally filled by the deposited Au in the sample shown in Figure 3a, whereas the gap between Si nanowires appears in the sample shown in Figure 3b. If the Si nanowire template was immersed into the plating solution while no potential was applied, then neither the Au particle nor the tube was found on the wall of Si template (Figure 3c). Therefore, the results shown in Figure 3 proved definitely that the deposition of Au in this experiment was because of electrochemical process, but not of electroless plating.

For the electrochemical deposition of metal onto macroporous Si, there are three typical deposition modes, which represent the deposition proceeding from pore bottom to pore opening [16,26,27], the deposition proceeding from the opening of pores [27], as well as the deposition occurring homogeneously on the entire surface of pore wall [16,17]. The homogeneous deposition occurs only for the deposition of less noble metal, whereas no radial growth on sidewall has been found for the noble metals so far. Therefore, macroporous Si has not yet been employed as a template for the electrochemical deposition of noble metal tube.

Noble metal tube is achieved with the use of Si nanowires as a template in this experiment. To explore the growth process of Au tube on Si nanowires template, the morphology of Au-deposited Si nanowires at the initial stage of deposition was investigated. For a deposition time of 5 min, the top (Figure 4b) and the middle (Figure 4c) parts of a Si nanowire are fully covered by Au layer, while the bottom part of a Si nanowires and the plateau between nanowires are loaded with isolated Au particles (Figure 4d). Especially, the density of Au particle on the plateau between Si nanowires is apparently lower than that on the bottom part of a Si nanowire. To further investigate the growth process of Au tube, the thicknesses of an Au tube at different sites apart from the root of an Au tube were measured, as shown in Figure 5a. It is shown that the top and middle parts possess almost the same thickness, while the root part of the Au tube is thinner than the remaining part of the tube. The morphologies of different parts of Au-deposited structures with short (Figure 4) and long (Figure 5) deposition times suggest that the growth of Au proceeds quasi-radially on the Si nanowires.

The mechanism of quasi-radial growth remains unclear so far. The difference between morphologies of Au on the top/middle parts (continuous film) and that of root part (isolated particles) of a Si nanowire might be induced by a mass transfer effect. Since the electrochemical deposition could take place everywhere on the exposed Si surface, the metal ions at the deposition front are consumed quickly once the electrochemical deposition starts. The subsequent supply of metal ions from bulk solution will be preferentially transported to the top/middle parts of the Si nanowires. In this case, the metal ions that can finally reach the root part will be much less because of the consumption of the top/middle part during the deposition, thus resulting in a thick top/middle part and a thin root part of the Au tubes.

The quasi-radial growth of Au on Si nanowires implies that the thickness of Au tube increases linearly with the deposition time, while the length of Au tube remains constant. The assumption has been confirmed by a series of control experiments (Figure 6). As shown by the TEM images of Au tube during different deposition times (Figure 6a-c), the thickness of wall in an Au tube does increase approximately linearly with the deposition time (Figure 6d). The results presented here suggest that the wall thickness of metal tube can be controlled by changing the deposition time, whereas the length of metal tube can be independently controlled via choosing Si nanowires template with a desired length. By further increasing the deposition time, the gap between Si nanowires is filled with the deposited Au. Consequently, the deposited Au evolves from tubular structure to a thick film with straight channels.

As mentioned above, by template-based metal-assisted chemical etching, the diameter of Si nanowires can be precisely controlled, and Si nanowires with diameters ranging from sub-10 nm to one micron have been achieved [21,23]. Accordingly, the inner diameter of an Au nanotube fabricated with Si nanowires as a positive template can be tuned in a wide range. Figure 7 shows a series of Au nanotubes with different inner diameters. Tubular structure with inner diameter as small as 45 nm was fabricated with Si nanowires from the AAO mask method (Figure 7c). The Si nanowires bend and stick together before the electrochemical deposition, and therefore bundles of Au tube are found (Figure 7c). The bending of nanowires and the formation of bundle are common phenomena for 1D nanostructure fabricated via solution-based method, due to surface tension force exerted on the nanowires during the drying of the sample [21,28]. The bending and bundling could be avoided or relieved by a supercritical drying process [24], thus potentially allowing the formation of isolated metal nanotube arrays with small tube diameters.

## Conclusions

In conclusion, Si nanowires have been employed as a template for the fabrication of noble metal tube by the electrochemical method. The growth of metal on Si nanowires proceeds quasi-radially, as suggested by SEM and TEM characterizations. This growth behavior enables precise control over the thickness of the deposited metal tube. Metal tubes with inner diameters ranging from 1 μm down to 45 nm are obtained by electrochemical deposition on the Si nanowires with preferred diameter.

## Abbreviations

AAO: anodic aluminum oxide; EDXA: energy dispersive X-ray analysis; HR-TEM: high-resolution TEM; PS: polystyrene; SAED: selected area electron diffraction; SEM: scanning electron microscope; TEM: transmission electron microscope.

## Competing interests

The authors declare that they have no competing interests.

## Authors' contributions

ZH carried out the etching experiments for Si nanowire templates and the electrodepositons, the SEM and TEM characterizations, as well as drafted the manuscript. LL participated in the electrodeposition and SEM characterization. NG carried out the RIE experiments during the fabrication of Si nanowires. All authors read and approved the final manuscript.
